# Health Beliefs and Perspectives of Parents Regarding Human Papillomavirus Vaccination in Kuwait: Qualitative Study

**DOI:** 10.2196/85438

**Published:** 2026-04-02

**Authors:** Ahmad Abuzoor, Md Shafiqur Rahman Jabin, Cor Jonker

**Affiliations:** 1 Faculty of Health Studies University of Bradford Bradford United Kingdom; 2 Department of Medicine and Optometry Linnaeus University Kalmar Sweden

**Keywords:** human papillomavirus, HPV, human papillomavirus vaccine, HPV vaccine, parents, Health Belief Model

## Abstract

**Background:**

After breast cancer, cervical cancer (CC) is one of the leading causes of female mortality. CC accounts for more than 7.5% of female cancer deaths worldwide. Human papillomavirus (HPV) is the most common sexually transmitted disease in women and the leading cause of CC in almost 99% of all CC cases. HPV vaccination could prevent up to 70% of HPV-related CC and 90% of genital warts. HPV vaccination is the bedrock of primary prevention and helps reduce the incidence and death rates of HPV-associated CC.

**Objective:**

The study aimed to understand the knowledge, health beliefs, and perspectives of Kuwaiti parents regarding HPV vaccination, with the goal of developing a health promotion policy and introducing a national immunization program in Kuwait.

**Methods:**

A total of 37 participants were evaluated using purposive sampling to select 20 (54%) participants for one-on-one semistructured interviews. We wanted to include both participants (male and female parents) with primary education (diploma or below) or secondary and higher education (bachelor’s degree and above). We had four categories (male parents/guardians with a diploma or below, male parents/guardians with a bachelor’s degree or above, female parents/guardians with a diploma or below, and female parents/guardians with a bachelor’s degree or above) with at least 5 participants in each category, which gave us 20 participants. Semistructured interviews were based on the Health Belief Model (HBM). The data were thematically analyzed using an inductive approach, generating themes through the theoretical framework of the HBM, and theme extraction analyses were managed on a semantic level.

**Results:**

We identified 7 main themes containing 20 subthemes. The seven themes were (1) knowledge and awareness about HPV infection and vaccination (3 subthemes); (2) perceived susceptibility, which is explained by the HPV infection effect based on sex (2 subthemes); (3) perceived barriers to HPV vaccination (8 subthemes); (4) perceived benefits (1 subtheme); (5) perceived severity (2 subthemes); (6) perceived efficacy (2 subthemes); and (7) cues to action (2 subthemes).

**Conclusions:**

The HBM framework is beneficial for Kuwait’s HPV vaccination campaign. The correlation between sexual intercourse and the HPV vaccine frequently adds complexity to the decision-making process about immunization. This study demonstrates that positive cues to action from health care practitioners and educational vaccination benefits can overcome perceived barriers among parents related to stigma and religion. It is essential to conduct more such research to guide the development of interventions aimed at promoting adoption of the HPV vaccine.

## Introduction

### Background

Human papillomavirus (HPV) is the most common sexually transmitted disease worldwide and is responsible for nearly all cases of cervical cancer (CC). Moreover, anogenital cancers occur in both men and women [1,2]. HPV 16 and 18 have been implicated in 70% of all CC cases in the world [3,4]. CC is the third-most common cancer among women in high-income nations and the second-most common in medium-income countries, after breast cancer. CC, in general, causes around 12% of all cancers worldwide and 18% of cancer among women in the Middle East [5]. CC is unlike many other malignancies and has excellent potential for primary prevention with HPV vaccination and secondary prevention with CC-screening methods, such as cytology (Papanicolaou [Pap] smear) and HPV testing, which enable early detection and treatment of precancerous lesions [6-8].

In Kuwait, the HPV vaccine is not included in the national vaccination program; however, it is accessible through the private health care sector [9]. At the end of 2024, the government health care center made the HPV vaccine available as an option. One significant barrier to CC prevention in Kuwait is the lack of a nationwide HPV vaccine and cervical cancer–screening program, with cytology being the most common screening technique [10,11]. Consequently, many CC cases are diagnosed at advanced stages, which hampers treatment and increases mortality.

Despite being a high-income country, Kuwait is one of the few nations without policies on CC vaccination and screening programs. Therefore, it is critical to identify the elements necessary for successful implementation of such a policy in Kuwait. The purpose of this study was to investigate parents’ perspectives, knowledge, and beliefs about HPV vaccination in Kuwait to develop an effective health promotion policy, based on analysis of data collected from semistructured interviews of 20 participants using questions based on the Health Belief Model (HBM). Each theme was analyzed based on the responses to questions on HPV and HPV vaccine knowledge and beliefs among Kuwaiti parents. The HBM was used as the theoretical basis for this study since it is a commonly used motivational framework for developing health education programs and supporting beneficial actions or behaviors that assist in lessening actual or perceived health concerns [12]. The Middle East has its own cultures and beliefs, much like the West and other parts of the world. The HBM is a framework that attempts to predict health-related behaviors based on specific belief systems (the Kuwait system).

### Study Aims and Objectives

The aim was to establish a health promotion policy by assessing Kuwaiti parents’ deep understanding and health beliefs regarding the HPV vaccine through an interview based on the HBM.

Toward this end, the study had the following specific objectives:

- Conduct semistructured interviews of Kuwaiti parents of children aged 12-17 years to determine what they know and believe about the HPV vaccine.
- Quantify the health beliefs and knowledge that are used to guide parents in deciding to vaccinate their children against HPV.
- To understand Kuwaiti parents’ knowledge of HPV and HPV vaccination by looking for gaps and ignorance in order to develop recommendations for health promotion to vaccinate against HPV.

## Methods

### Study Design

This study used a qualitative approach by conducting face-to-face semistructured interviews with participants. Data collection involved open-ended questions, targeting a purposive sample of Kuwaiti parents with children aged 12-17 years. Thematic analysis was used to analyze the data.

### Theoretical Framework

This study used the HBM as a theoretical framework because of its articulate, measurable constructs—perceived susceptibility, perceived severity, perceived benefits, perceived barriers, cues (to action), and self-efficacy—which can be easily mapped onto parental cognition and can be operationalized in survey and interview measures [13,14]. The interview questions were designed to align with the HBM’s components, aiming to understand how parents perceive HPV vaccination in terms of perceived susceptibility (questions about HPV transmission and cancer risk), perceived severity, perceived benefits and barriers (questions about the reasons for or against vaccination), self-efficacy, and cues to action. The HBM identifies barriers to behavior change. In this study, the HBM was used to explain participants’ (parents) health behavior. For example, the participant was asked about the perceived benefits of HPV: “What are the health benefits that lead you to think about getting the HPV vaccination for your child, or not, in case it is available?” (Multimedia Appendix 1).

Nevertheless, the HBM has been identified to have shortcomings: it focuses on individual cognitions, and in most instances, it explains only a modest level of behavioral variance in complex behaviors in public health [15]. We used part 1 of the framework to explore the principle of consent in this model through the interview questions.

### Study Site and Sampling

Qualitative research typically uses a smaller sample size than quantitative studies, because instead of applying the findings to an entire population, it attempts to obtain deeper knowledge and meaning [16]. A purposive sampling strategy was used to select 20 parents/guardians of 12-17-year-old children from intermediate-level schools in Kuwait. Both male and female parents were eligible to participate in the study.

This study also explored the different information and themes in male responses compared to those in female responses. Both sexes were included, with primary, secondary, or higher education. The criteria used to select a sample based on maximum variation were sex and educational attainment. We had four categories (male parents/guardians with a diploma or below, male parents/guardians with a bachelor’s degree or above, female parents/guardians with a diploma or below, and female parents/guardians with a bachelor’s degree or above) with at least 5 participants in each category, resulting in at least 20 participants. Parents could opt in to the qualitative study at the end of the quantitative study [17]. Based on the aforementioned criteria, the individuals who were excluded were also sent an explanation of why they had not been included in the study.

A total of 18 schools were selected for the study, with 6 (33%) schools chosen from each of the three educational regions. Since the study focused on parents with school-age children, the sample was based on selecting participants who positively responded to the questionnaire and purposefully choosing respondents who represented the aforementioned four categories. All participants were recruited on a rolling basis. We used purposive sampling to select parents of children from the 18 schools. Purposive sampling ensures that participants selected have useful and relevant knowledge, and it is often used to optimize resource use while collecting the maximum amount of valuable data [18]. In addition, participants are chosen based on the characteristics of the population, needs, and study design. Purposive sampling is one of the most popular nonprobability sampling methods for obtaining data in a particular field and can be especially useful for qualitative research. This way researchers can focus on choosing participants who fit the research aim and are most likely to contribute to knowledge of the phenomenon in question. Purposive sampling makes sense if the information-laden cases provide nuance and insight, and qualitative studies that want to dive deeper or more complicated issues could benefit from purposive sampling.

Once identified as being eligible for the study, participants were sent an interview invitation based on their answers to the last question in the questionnaire, “Would you like to participate in an interview?”

### Ethical Considerations

Ethical considerations were addressed via an official letter from the Kuwait Ministry of Health (MOH; #1345/2020) and the Ministry of Education (reference 25914), and ethical approval was also obtained from the Ethical Committee of the University of Bradford (E844). Participants who accepted the interview invitations ascertained their interest by signing a consent letter that provided them with an overview of the study, in addition to an information sheet (Multimedia Appendix 2). The information sheet clearly stated that participation in the study was for research purposes only.

To obtain the consent to participate, participants were given time (about 1 week) to make their own decision and sign the consent letter. Interviews were scheduled at convenient times and locations. Given the sensitivity of Kuwaiti culture, the interviews were audio-recorded with the participants’ permission; if any participant refused to be recorded, only notes were taken. To minimize any potential misunderstanding by participants during the interviews, the researcher ensured that participants had an opportunity to elaborate on unclear points. In addition, the researcher read back the notes made during the interview to double-check their accuracy and provide the participant with an opportunity to explain or elaborate. This made the data more trustworthy and also made sure that the perspectives and opinions of the participants were captured accurately.

Data will be preserved for 5 years and archived within the University of Bradford, and no medical experiments, medicines, biopsies, or other interventional testing will be conducted in the future.

### Data Collection

Research data are essential to reaching meaningful conclusions because they reveal participants’ perspectives. Data collection is a process of taking actions to tackle research questions and obtain information [19]. Semistructured interviews are particularly useful in helping to get a sense of the context and what is going on with a descriptive study and understand the attitude of participants [20]. In this study, before the interviews, participants received an open-ended interview guide with questions based on parents’ health beliefs regarding HPV vaccination. This protocol made sure that the discussions were still anchored in the research aim but gave room to all researchers to explore what they had learned and felt.

The interview questions were written in English and then translated into Arabic, Kuwait’s official language. The interviews and data collection were conducted in Arabic and transcribed. The thematic analysis was conducted in Arabic and later translated into English by an authorized translation center to avoid losing any insight from the answers. The official translation office verified the translations. The transcripts were read and re-read to obtain a comprehensive perspective on the data; subsequently, the transcripts were compared to the original notes, and the data were presented. Cost and time elements were also obtained.

Each interview lasted 30-60 minutes, allowing participants to express their views on HPV vaccination. Once at the interview site, each participant was administered a demographic questionnaire to gather information such as age, marital status, and education level (already in the questionnaire but to ensure this information was correct). To ensure the effectiveness of the final interviews, a pilot study was conducted with 5 parents before the main data collection. This was a pretest phase to determine whether the interview questions and study resources were helpful and clear, as well as how much time was required for all the steps. The pilot study also helped detect potential difficulties that could have arisen during the interviews and let the researcher polish up the process and the quality of data collection in general.

### Data Analysis

After conducting the semistructured interviews, the recordings were transcribed verbatim to accurately represent participants’ answers. The data were filtered using themes from the open-ended questions. Such unravelling and corralling of data to find commonalities and patterns helped us read, explain, and understand the data [21,22].

This study used thematic analysis, a common qualitative analytical technique. It involves spotting, interpreting, and summarizing patterns or themes in qualitative data to produce a detailed and refined picture of the phenomenon under consideration. Deductive analysis is based on assumptions about already present theories or frameworks, while in inductive analysis, themes and codes appear spontaneously from the data. We used inductive thematic analysis to identify codes, themes, concepts, and categories within the interview data. This methodology provided us with the flexibility to ask parents about their health beliefs and knowledge regarding HPV vaccination, without having to follow set rules; this gave meaning to the data through theme selection and grouping. This process ensured a detailed and complete appreciation of participant perception, which was used to meet the study’s aims [23]. We used an iterative analytical process, in which three to four transcripts were reviewed independently to identify key themes. Subsequent sets of interviews were similarly assessed to clarify core themes. We also developed and applied codes that explained the categorization of the data. The HBM was used to structure the interviews. Therefore, the interview outcomes were categorized under HBM components (perceived susceptibility, perceived severity, perceived benefits, perceived barriers, perceived self-efficacy, and cues to action). A code was created, and themes evolved because we used that as a structure to try to analyze what the parents said and the questions already focused on these components. The open coding of the transcribed interviews helped increase understanding of the data’s meaning and significance. Manual open coding involved line-by-line repeated reading and coding to identify themes and subthemes. Two supervisors conducted identical evaluations to ensure the validity of this procedure. Any differences of opinion were settled by reviewing the entire thematic analysis process (codes, themes, and subthemes) and then reaching a consensus to make the final decision about the themes that evolved.

## Results

### Themes and Subthemes

The interview transcripts were analyzed by reading the obtained data line by line. This process continued, and 221 codes were created. The initial codes from each interview were then categorized, given new titles, and assigned to distinct themes and subthemes. These themes and subthemes were combined if duplicates were found in order to eliminate any conceptual repetition. There were 7 main themes, with a total of 20 subthemes, that explored HPV and HPV vaccine knowledge and health beliefs among Kuwaiti parents. Table 1 provides a list of the final themes and subthemes, followed by example quotations for a few subthemes.

**Table 1:** Themes and subthemes identified in the study.

Theme number	Theme	Subthemes
1	Knowledge and awareness about HPV^a^ infection and vaccination	Subtheme 1: parents’ knowledge and awareness about the HPV vaccine Subtheme 2: importance of the HPV vaccine for both sexes Subtheme 3: ways of transmitting HPV and the effects of factors
2	Perceived susceptibility	Subtheme 1: relationship of HPV infection and sex Subtheme 2: parents’ perspectives on getting an HPV infection
3	Perceived barriers	Subtheme 1: lifelong stigma, social customs, and negative HPV vaccine uptake Subtheme 2: social and familial influences regarding HPV vaccination and decision-making about immunization Subtheme 3: religious and societal influences regarding HPV and HPV vaccination on decisions Subtheme 4: fear of complications from the vaccine Subtheme 5: previous bad experiences with vaccines and medications Subtheme 6: cost of the HPV vaccine Subtheme 7: trust in the HCS^b^, confidence in medical staff, and satisfaction with the vaccination Subtheme 8: availability of authentic and reliable information about the HPV vaccine
4	Perceived benefits	Subtheme 1: benefits of getting an HPV vaccine as protection from and prevention of the virus and related cancer
5	Perceived severity	Subtheme 1: fear of HPV infection being a cause of cancer or severe disease Subtheme 2: viral disease that affects the skin or appears in the form of a wart
6	Perceived efficacy	Subtheme 1: parents’ preferences and experiences influencing decisions regarding HPV vaccination Subtheme 2: availability of the vaccine in private hospitals and lack of availability in government hospitals
7	Cues to action	Subtheme 1: role of the government and the MOH^c^ Subtheme 2: awareness through campaigns, the media, HCPs^d^, and schools

^a^HPV: human papillomavirus.

^b^HCS: health care system.

^c^MOH: Ministry of Health.

^d^HCP: health care practitioner.

We used HBM concepts in this analysis to align and extend the themes with the HBM framework. The HBM provided a theoretical framework to explore health beliefs among parents in Kuwait and the associated knowledge.

### Theme 1: Knowledge and Awareness About HPV infection and Vaccination

#### Subtheme 1: Parents’ Knowledge and Awareness About HPV and the HPV Vaccine

Some participants had some basic knowledge of HPV infection. For example, a male parent with a bachelor’s degree said:

It is a type of viral disease that affects the skin in the form of small granules and may cause cancer.

In contrast, other participants had no knowledge or limited knowledge of HPV infection and its vaccine. For example, a female parent with a diploma said:

I do not know about HPV and [have] no awareness about it. I do not know well about HPV vaccine and how it works. I have no idea. Is it for girl or boys to give it?

#### Subtheme 2: Importance of the HPV Vaccine for Both Sexes

Some participants mentioned that the vaccine is essential to both sexes. A female parent with a bachelor’s degree said:

The answer is that both sexes are the same. There is no difference between girls and boys in terms of vaccination priority.

#### Subtheme 3: Ways of Transmission of HPV and the Effects of Factors

Participants highlighted that the major reason for HPV infection is illegitimate relationships with several partners. A female parent with a bachelor’s degree said:

It is an infection transmitted through sexual intercourse.

Theme 1 and its subthemes revealed that participants were at different levels of knowledge regarding HPV and its vaccine regardless of their level of education or sex.

### Theme 2: Perceived Susceptibility

#### Subtheme 1: Relationship of HPV Infection and Sex

Some participants associated HPV infection with women and girls. These participants were told that the perceived susceptibility to HPV is linked to being a woman, which would result in HPV in female individuals more than in male individuals. For example, a male parent with a diploma said:

A virus that infects a woman’s reproductive system...

Another female parent with a bachelor’s degree said:

Maybe it will affect pregnancy if women take it before marriage.

However, some participants were aware that HPV affects both sexes and may have devastating consequences for both sexes. A male participant with a doctorate said:

A virus that infects a woman or a man as a result of multiple illegal sexual relations...

#### Subtheme 2: Parents’ Perspective on Getting an HPV Infection

Some of the participants said that their children can get an HPV infection but at a lower percentage. One female parent with a high school degree said:

I do not know the chances because life will change a few years later, and I cannot tell you the behavior that the boy or girl will have in [the ] future.

Most of the participants said they did not know whether their children will get an HPV infection. They did not give any reasons, which was not related to the sex or education level of the parents:

I don’t know the chance to get this virus.

In contrast, other participants provided different reasons for why they believed that they were not at risk for HPV infection. For example, a participant with a bachelor’s degree thought there was no chance of an HPV infection because he trusted his children:

There is none because customs and traditions dictate that you be bound to one person, and I trust my children.

Only one female parent with a bachelor’s degree believed in her children’s perceived susceptibility to infection and felt the chances of getting infected are high based on many things changing these days.

In these days, [there] will be a high chance of being infected.

Theme 2 and its subthemes show that some parents feel that HPV affects only female individuals, while others say it could affect both sexes. In addition, some parents mentioned that there was no chance of their children getting an HPV infection, while others felt that their children might get infected.

### Theme 3: Perceived Barriers

In this study, many subthemes regarding perceived barriers fit the HBM. Parents of both sexes and all education levels reported several barriers.

#### Subtheme 1: Lifelong Stigma, Social Customs, and Negative HPV Vaccine Uptake

Participants highlighted that they might be reluctant to vaccinate their children against HPV because of the stigma associated with it. One female participant with a diploma refused to vaccinate her daughter, feeling that it was unnecessary because her daughter was unlikely to contract the virus due to traditions:

We are a conservative society with social customs and traditions, where most married people are only related to one person. It may be a lifelong stigma for her, and she will not marry after that.

One of the male participants with a doctorate mentioned that a feeling of shame may be one of the barriers that can affect him when he decides to vaccinate his children:

It may negatively affect the girl’s psychology in terms of her feeling ashamed, given that we are a conservative society, so they can understand that this wrong.

#### Subtheme 2: Social and Familial Influences Regarding HPV Vaccination and Decision-Making About Immunization

One of the barriers discovered in this study was related to cultural and familial issues. Some participants stated that in Kuwaiti society, the decision to receive the HPV vaccine is often associated with promiscuity. A female parent with a diploma said:

I will worry regarding the HPV vaccine; vaccination will not be widely accepted in Kuwaiti society. Therefore, I will worry and think a lot before giving it to my daughters. It is new for Kuwaiti society, as it only exists in the West.

Another female parent with a bachelor’s degree said:

There are no factors that encourage me to give it [HPV vaccine] to my sons because this disease results from illegal relationships.

#### Subtheme 3: Religious and Societal Influences Regarding HPV and HPV Vaccination on Decisions

Religious beliefs and the idea that Islamic norms prevent risky sexual behavior were often mentioned as reasons to consider HPV vaccination unnecessary or unsuitable. Parents commonly said that following Islamic law and local customs protects against behaviors that could cause HPV infection, so they felt the vaccine was more fitting for other communities.

One father with a master’s degree expressed this opinion clearly:

We follow Islamic law, and our customs and traditions preserve, preserve, capture these, preserve these points. There are no illegal relationships, and there are not many homosexuals.

#### Subtheme 4: Fear of Complications From the Vaccine

Another perceived barrier identified was the fear of complications from HPV vaccination. Therefore, some of the participants hesitated to get their children vaccinated. Participants also highlighted that the HPV vaccine might have side effects. A male parent with a bachelor’s degree said:

I am afraid of the side effects of this vaccine when I decide to vaccinate my daughters.

#### Subtheme 5: Previous Bad Experiences With Vaccines and Medications

Participants highlighted that people’s bad experiences with COVID-19 vaccines had made them reluctant to get their children vaccinated against HPV. A male participant with a master’s degree said:

This vaccine could prevent [HPV], but as far as I know, the spread that happened with previous vaccines [COVID-19]...I expect this vaccine to be harmful or useless or have side effects and complications that are more [harmful] than useful.

#### Subtheme 6: Cost of the HPV Vaccine

Another perceived barrier to HPV vaccination was the cost of the vaccine. A female parent with a diploma said:

I found it [HPV vaccine] in private hospitals, but the price of the dose is approximately 35 Kuwaiti dinar

US $110

#### Subtheme 7: Trust in the Health Care System, Confidence in Medical Staff, and Satisfaction With the Vaccination

Male parents with high school education highlighted that doctors generally do not recommend the HPV vaccine in routine clinical practice:

Also, it is not recommended by doctors, as I visited hospitals and polyclinics a lot for my sons, but I have yet to hear anyone speak to me about this vaccination.

#### Subtheme 8: Availability of authentic and reliable information about HPV vaccine

Parents commonly noted a lack of reliable, locally relevant information concerning HPV and the HPV vaccination. This information vacuum left individuals unsure about the benefits and risks of vaccines, prompting some to rely on informal or online sources that did not fully reassure them.

A male participant with bachelor’s degrees participant expressed:

It makes me discouraged that there is no information about this vaccination. Therefore, we do not know how effective it is, and therefore, we will not take this vaccination.

Another father attributed his reluctance directly to the lack of existing studies:

It is possible that I will not give my daughters the vaccination, as there are no studies that I have read before that, especially in Kuwait.

Theme 3 and its subthemes explain the barriers in parents’ minds regarding HPV vaccine uptake.

### Theme 4: Perceived Benefits

Perceived benefits of health-promoting behaviors refer to an individual’s subjective perception of the beneficial effects and rewards associated with engaging in those behaviors. In this study, although some participants had a superficial knowledge of HPV or had incorrect ideas about their children’s susceptibility to the virus, they noted the positive benefits of engaging in preventive actions.

#### Subtheme 1: Benefits of Getting an HPV Vaccine as Protection From and Prevention of HPV Infection and Related Cancer

The prevention of cancer linked to HPV is one of the HPV vaccine’s main benefits. Participants highlighted that due to the perceived benefits of the HPV vaccine, they might vaccinate their children. A female participant with a bachelor’s degree mentioned that if the HPV vaccine has benefits, she would vaccinate her children:

[It will] encourage me to vaccin[ate] my children if it has a benefit for my children.

Male participants with high school education mentioned the prevention of cancer as a benefit:

The vaccine, in general, is preventing disease; maybe, anyone has this virus. But if receiv[ing] the vaccine means that will decrease cancer in the body or prevent it, this is a very positive thing.

### Theme 5: Perceived Severity

Perceived severity relates to the way in which participants believed that HPV infection may severely impact the quality of life of their children. Participants highlighted the possible severe health ramifications of HPV infection, including CC, genital warts, and skin disease.

#### Subtheme 1: Fear of HPV Infection Being a Cause of Cancer or Serious Disease

One male parent with a doctorate mentioned that HPV and cancer are related:

There is a close relationship between HPV and cancer, as it is possible, in a large percentage, for this virus to turn into cancer. Also then, it can turn into CC in women and penis cancer in men.

One female parent with a bachelor's degree said that HPV “causes CC.”

#### Subtheme 2: Viral Disease That Affects the Skin or Appears in the Form of a Wart

Some participants mentioned that HPV infection can affect the skin in the form of warts. A male parent with high school education said:

A virus that infects humans through skin-to-skin contact, either through sexual intercourse or skin to skin

Another male participant with a bachelor’s degree said:

[Appears on] the skin in the form of small granules and may cause cancer

### Theme 6: Perceived Efficacy

#### Subtheme 1: Parents’ Preferences and Experiences Influencing Decisions Regarding HPV Vaccination

Self-efficacy relates to a person’s belief in their ability to change or perform a behavior effectively. Participants highlighted that parents’ preferences and experiences can influence their decision to vaccinate their children. One male parent with a master’s degree mentioned that he will vaccinate his children when he sees an infection among people:

The factors that encourage me to vaccinate my son are that I see many infections among people and that I see them with my own eyes.

#### Subtheme 2: Availability of the Vaccine in Private Hospitals and Lack of Availability in Government Hospitals

A male participant with a bachelor’s degree added:

I read that there is a vaccine for this virus but outside Kuwait, and I have not heard of it in Kuwait.

### Theme 7: Cues to Action

#### Subtheme 1: Role of the Government and the MOH

Cues to action are unique triggers that encourage individuals to make changes that promote their health. Participants engaged in a discussion of the significance of external cues that prompt action. They said that they would take action if health care practitioners (HCPs) strongly recommended vaccination. A male participant with a doctorate highlighted the role of the MOH and the government:

The factors that encourage me to take my child for vaccination: First, that this vaccination should be included in the vaccination schedule of the Kuwaiti MOH, where I will be very reassured.

A female participant with a diploma said:

I do not know anything about it, and [I have] not been made aware of it by MOH, but if MOH advises us to vaccinate, I will give it to my daughter after they explain the benefits and side effects of this vaccine.

#### Subtheme 2: Awareness Through Campaigns, the Media, HCPs, and Schools

Another example of cues to action is a campaign encouraging people to vaccinate against HPV. A male participant with high school education said:

Awareness campaigns must be made for parents to encourage them to vaccine their children. We trust MOH if they advise it.

According to the HBM, a cue, or trigger, is necessary to encourage people to adopt health-promoting attitudes. Participants highlighted that advice and/or prescriptions from doctors can help parents decide about HPV vaccination. A female parent with high school education said:

It encourages me that doctors recommend it during a regular visit to a polyclinic or hospital or when asking about that HPV vaccination.

## Discussion

### Principal Findings

In this study, overall, there was variability in knowledge and awareness regarding HPV. Some parents had no knowledge, while others had only basic knowledge and awareness about HPV, its transmission, and the HPV vaccine. A study [24] conducted in Indonesia showed a lack of knowledge about HPV and sexually transmitted diseases among parents regardless of sex. Another study [25] conducted in Australia also reported a lack of knowledge about HPV among parents; although the majority of parents had heard about HPV, only two had strong information about HPV and how it is transmitted [25].

This study identified many barriers to HPV vaccine uptake. The main obstacles are stigma, religion, societal influences, risk and fear of vaccine complications, cost, and the availability of authentic and reliable information about the HPV vaccine. Religious and cultural reasons were found to be some of the main barriers in a study [26] conducted among Syrian mothers in 2011; results showed that cultural influences lead mothers to perceive that their daughters are not at risk for HPV and, therefore, do not need vaccination [26].

Many studies have shown the benefits of the HPV vaccine from parents’ perspectives. Parents have explored the benefits of the HPV vaccine in preventing CC and believe that it would reduce the impact of the infection [27-30]. In this study, we observed low perceived susceptibility regarding HPV infection, as half of the parents enrolled did not know whether their children can get an HPV infection. Prior studies have established that the perceived susceptibility to developing HPV infection among different communities is low. In one study [31] in Saudi Arabia, the parents said their children were not at risk for infection, so they would not vaccinate them.

Perceived severity is one of the HBM concepts that explores the seriousness of infection or disease. A different community in Indonesia mentioned that there is a relationship between HPV and cancer, but few participants noted it [24]. Another study [32] in Saudi Arabia showed that HPV infection is the main cause of cancer. Our findings reveal perceived efficacy as another HBM concept based on parents’ views. Previous studies have addressed this issue; a mother mentioned that a shift in the young generation’s thinking encouraged her to vaccinate her children [33]. In another study [34], a participant mentioned that her daughter was too young for the vaccine and that she would wait for her to be older.

Cues to action are another HBM concept that explains to parents how to take action for their health. Our findings showed how parents would take action to vaccinate their children against HPV. Previous studies have found that HCPs’ recommendations can motivate parents to accept the HPV vaccine by advising them and explaining about HPV [35]. Similarly, if doctors recommend the HPV vaccine, parents can make an informed decision based on clear and trusted information [36].

### Strengths and Limitations of the Study

One of the study’s strengths was the use of the HBM, which has three strengths: a comprehensive framework, individual focus, and practical application. The HBM provides a comprehensive framework for understanding health-related behaviors by examining a wide range of factors that affect people’s behavior. The model’s simplified health-related components support ease of implementation, application, and testing. The HBM is the best model for health promotion [37]. The advantage of this qualitative research is that it can provide a comprehensive description of participants’ opinions about an HPV vaccine, thereby strengthening our assessment of the reality of HPV vaccine uptake in the context of their deepest health beliefs regarding HPV infection and its vaccine [38].

One of the limitations of this study is that the interviews were unavoidably restricted to just Kuwaiti citizens because no non-Kuwaiti citizens volunteered. This limits the generalizability of the findings to the wider population residing in Kuwait. In addition, the study was conducted exclusively in government schools and did not collect data from private schools, with the majority of pupils being non-Kuwaiti residents. Therefore, these results are unlikely, given the knowledge and health beliefs of all parents in Kuwait. Furthermore, given that we examined the delicate subject of HPV and sexual health, it is plausible that the participants abstained from disclosing their experiences because of concerns regarding adhering to social standards. The findings of this research may not be applicable to the entire population of parents in Kuwait. All parents refused to permit audio-recording during the interviews. The researchers, who are Kuwaiti, realized that the participants were cautious and expected this. As a result, the researchers took notes during each interview and transcribed them. This allowed the researchers to note the most important points made by the participants, although the precise way in which the participants made those points was somewhat lost.

As the data for the study were collected during the COVID-19 pandemic or near the end stage of COVID-19, all recommended precautionary measures related to the pandemic were taken during data collection. However, the precautionary measures taken due to COVID-19 may have had some effect. For example, as the COVID-19 vaccines were new, many people hesitated to take them; similarly, participants may fear the impact of the HPV vaccine since it is new to most participants. Some complications have arisen due to the COVID-19 vaccine, which may also occur with the introduction of a new vaccine, such as the HPV vaccine.

### Conclusion

Over 90% of HPV-caused cancers (mainly CC) may be avoided with the HPV vaccine. HPV vaccination serves as the principal preventive measure against CC. Positive cues to action from parents after advice from the MOH and HCPs about the benefits of vaccination can overcome perceived barriers among parents related to stigma and religion. Most parents have been motivated to protect their children and prevent disease. The correlation between sexual intercourse and the HPV vaccine frequently adds complexity to the decision-making process about immunization. National health care systems and HCPs can enhance the importance of getting the HPV vaccine and explain why vaccination is recommended by giving clear and timely information. They must alleviate parental concerns over vaccination efficacy and safety, while also considering diverse religious, social, and cultural sensitivities, as well as varying educational backgrounds. The findings from this study demonstrate the potential of using the HBM to overcome perceived barriers to HPV vaccine uptake with culturally appropriate interventions and accurate information.

## Data Availability

All supporting data are reported in the article.

## Appendix

Multimedia Appendix 1 Semistructured interview questions.

Multimedia Appendix 2 Consent form.

## Abbreviations

CC: cervical cancer
HBM: Health Belief Model
HCP: health care practitioner
HPV: human papillomavirus
MOH: Ministry of Health
