# Supplementary material for: Health Beliefs and Perspectives of Parents Regarding Human Papillomavirus Vaccination in Kuwait: Qualitative Study
Source: JMIR Form Res. 2026 Apr 2;10:e85438. doi: 10.2196/85438 (PMC13087555; doi:10.2196/85438)
Supplement: Multimedia Appendix 1 [file formative_v10i1e85438_app1.docx]

### Appendix 1: Health beliefs perceptions of parents towards HPV vaccination: a qualitative study in Kuwait (Interview Questions).

**Part 1: Knowledge about HPV/HPV vaccine:**

1. Can you tell me what you know/understand about HPV?

Possible prompts:

- How is HPV spread?
- What do you know about the relationship between HPV and cancer diseases?

1. What do you know/understand about the HPV vaccine?

Possible prompts:

- What do you think about the importance of HPV vaccine prevention from cancer diseases?

1. What do you know about the issues that could predispose individuals to cervical cancer?

**Part 2: Parents’ understanding about the HPV vaccine:**

1. What are the general factors that encourage you, or discourage you, from taking your child to get a vaccination against HPV?
2. How is the government policy towards the HPV vaccine influencing your attitude towards the vaccination for your child?
3. Is there anything that worries you about the HPV vaccination?

Possible prompts:

- How have these worries affected your attitude towards the vaccination?

**Part 3: Parents and health beliefs regarding HPV/HPV vaccine:**

1. What are the health benefits that lead to you thinking about taking the HPV vaccination, or not, in case it's available?

Possible prompts:

- Would you feel more comfortable vaccinating a son or a daughter?
- How do you think gender matters in HPV vaccine?
- What do you think are the chances of HPV infection for your child?
- How do you think the HPV infection can or cannot get a serious disease for your child?
- Can you explain the benefits and drawbacks of the HPV vaccine in your view?
- What are the barriers that you could face when deciding to vaccinate your child in your opinion?
- Can you tell me what level of protection is offered by the HPV vaccine against transmitted sexual infection?
- Can you explore the related to the benefits of receiving the vaccine?
- What is the intrinsic motivation in taking the HPV vaccine if it is available?

1. Do you have anything else you would like to add?

Thank you for your answers.
