# Supplementary material for: Health Beliefs and Perspectives of Parents Regarding Human Papillomavirus Vaccination in Kuwait: Qualitative Study
Source: JMIR Form Res. 2026 Apr 2;10:e85438. doi: 10.2196/85438 (PMC13087555; doi:10.2196/85438)
Supplement: Multimedia Appendix 2 [file formative_v10i1e85438_app2.docx]

### Appendix 2: Consent form (interview)

**Title of the project:** Attitudes and health beliefs of parents towards HPV vaccination: a qualitative study in Kuwait.

Name of Researcher: Ahmad Abuzoor

Contact details**:** *Tel: +965 97943900 / +44(0) 7365439397*

*email:* [arsabuzo@bradford.ac.uk](mailto:arsabuzo@bradford.ac.uk)

**Please initial all boxes if you agree**

I confirm that I have read and understand the information

sheet describing the purpose of this study and have had the

opportunity to ask questions about the study.

I understand that a recorded copy of the interview will be transcribed

and will be analysed by the researcher for the purposes

of a postgraduate research study.

I understand that sections of these transcripts may be quoted

in the researcher’s thesis and in other outputs but anonymity

will be kept throughout.

I agree to participate in an interview with the researcher.

I understand that my participation is voluntary without

legal rights being affected.

I understand the interview will be recorded.

I understand if I refuse the recording, only notes will be taken.

Name of participant Date Signature

_____________________________

_______________
